# Individual differences in dominance-related traits drive dispersal and settlement in hatchery-reared juvenile brown trout

**DOI:** 10.1038/s41598-021-86613-4

**Published:** 2021-03-31

**Authors:** Jorge R. Sánchez-González, Alfredo G. Nicieza

**Affiliations:** 1 Department of Biology of Organisms and Systems, University of Oviedo, 33006 Oviedo, Spain; 2 Department of Animal Science - Wildlife Section, University of Lleida, 25198 Lleida, Spain; 3 Research Unit of Biodiversity (UMIB:UO-CSIC-PA), Campus de Mieres, 33600 Mieres, Spain

**Keywords:** Ecology, Behavioural ecology, Ecophysiology

## Abstract

Effective management of exploited populations is based on an understanding of population dynamics and evolutionary processes. In spatially structured populations, dispersal is a central process that ultimately can affect population growth and viability. It can be influenced by environmental conditions, individual phenotypes, and stochastic factors. However, we have a limited knowledge of the relative contribution of these components and its interactions, and which traits can be used as reliable predictors of the dispersal ability. Here, we conducted a longitudinal field experiment aimed to identify traits which can be used as proxy for dispersal in juvenile brown trout (*Salmo trutta* L.). We measured body size and standard metabolic rates, and estimated body shapes for 212 hatchery-reared juvenile fish that were marked with individual codes and released in a small coastal stream in northwest Spain. We registered fish positions and distances to the releasing point after 19, 41, 60 and 158 days in the stream. We detected a high autocorrelation of dispersal distances, demonstrating that most individuals settle down relatively soon and then hold stable positions over the study period. Body size and fish shape were reliable predictors of dispersal, with bigger and more robust-set individuals being more likely to settle closer to the release site than smaller and more elongated fish. In addition, the analysis of spacing and spatial patterns indicated that the dispersal of introduced fish could affect the distribution of resident conspecifics. All together, these results suggest that stocking programs aimed to the enhancement of overexploited populations at fine spatial scales can be optimized by adjusting the size and shape of the introduced fish to specific management targets and environmental conditions.

## Introduction

Dispersal is a central issue for the understanding of evolutionary processes and population dynamics^1,2^, with profound implications for current spatial distributions, local population densities, demographic dynamics, and the genetics of spatially-structured populations^3–5^. Moreover, dispersal processes are important because of their implications for forecasting animal distributions and for robust decision-making in the areas of resource management and conservation of biodiversity^6^. Identifying the drivers of dispersal can be difficult because dispersal events may depend upon extrinsic and intrinsic factors. Intrinsic factors can arise from consistent individual differences and extreme competitive asymmetries, or if dispersal ability is under genetic control^7–10^. On the other hand, in absence of inter-individual differences, we might expect dispersal to be a random process mainly determined by extrinsic, and eventually, fortuitous factors (environment-dependent dispersal). In this context, it may be worth seeking consistent suites of traits that define a ‘dispersal syndrome’^8–11^. For instance, concurrent changes in physiological, morphological and behavioural traits can exert an enormous influence on dispersal^12^.

In salmonid fishes, seasonal or unpredictable fluctuations in flow regimes affect dispersal directly by increasing drift during floods^13^ or via nutritional stress and competition for food during drought periods. Intense competition for resources has been often considered to be a driver of dispersal, and the likelihood and extent of the dispersal process can be affected by dominance status and individual aggressiveness^14^. Previous studies have evidenced positive relationships between metabolic rate and competitive ability^15^, dominance^16^, aggressiveness^17^, or developmental pathways^16,18^, and also between body shape and aggressive behaviour^19^. More aggressive individuals are expected to become dominant, gain access to better feeding positions, grow faster, and thus to establish territories, whereas inferior competitors could be displaced away from their natal areas^14^. Therefore, body size, and size-dependent physiological and behavioural traits (e.g., metabolic rate, boldness) are obvious candidates to find good proxies for dispersal propensity^20^. However, despite the known connections between shape, metabolic rate, size and competitive ability and dominance, the relative contribution of these traits to the dispersal process remains largely unexplored.

In some species, the process through which immature individuals depart their birth sites (‘natal dispersal’ in a strict sense) occurs through a series of successive movement and residency periods that can be associated with behavioural and life-cycle transitions or changes in the environmental conditions^21,22^. These bouts of movement after the initial depart from the birthplace can be defined as secondary dispersal^22^. The operating definition of secondary dispersal is useful because it can be applied to natural populations but also to the case of individuals introduced in a novel environment, regardless of they were translocated from other natural populations or artificially reared for population supplementation. Here we were aimed to investigate the drivers of secondary dispersal and settlement in a freshwater fish (brown trout, *Salmo trutta*) whose populations are largely subjected to invasive management (e.g., exploitation, stocking, and introduction in novel environments) by combining physiological and morphometric data with a longitudinal field experiment maintained over more than five months. In this context, we explore whether body size, metabolic rate, and body shape can be used as predictors of dispersal and conform a dispersal syndrome, which may be helpful for designing efficient stocking programs. Juvenile salmonids aggressively defend territories and establish dominance hierarchies^14,23^, which can influence dispersal^14^. We hypothesized that individuals with a higher specific (i.e., size-independent) metabolic rate, a more robust (deep-bodied) shape and a larger size are more likely to hold territories^14^, whereas fish with lower metabolic rates, elongated bodies, and smaller size would be forced to disperse (the ‘defender’ hypothesis). Alternatively, individuals with a higher metabolic rate, more robust shape or larger size could be more prone to disperse^24^ because it can promote the exploration and settlement in new, more favourable grounds, since highly competitive individuals might overcome priority effects (the ‘explorer’ hypothesis).

## Results

### Individual variation of body shape

**Figure 1:**
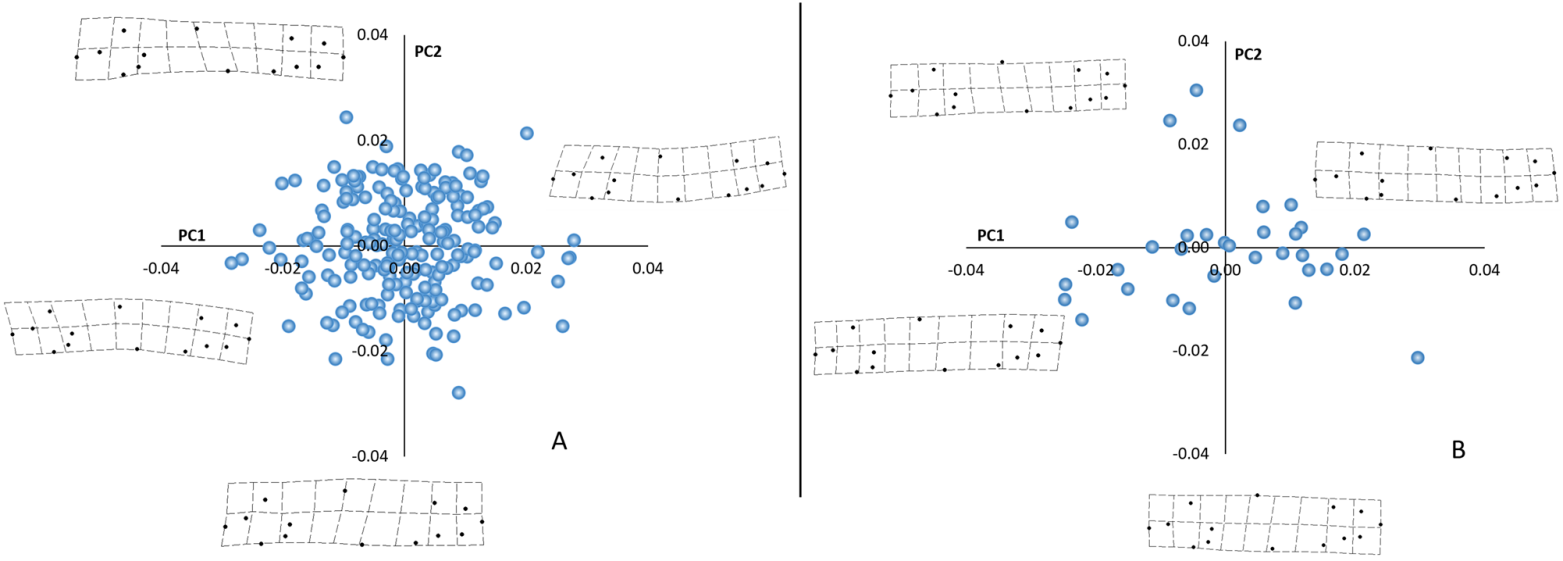
Scores of the PCAs performed on the variances-covariances matrix for the morphological variables. (**A**) PCA for the experimental individuals before release in the Santianes River, and (**B**) PCA for experimental fish recaptured on February, after several months in a natural stream. *PC1* negative scores are lower positioned peduncles, *PC2* negative scores correspond to deep-bodied and more robust shapes.

In late August (before release at the Santianes River), the first three principal components of a PCA on morphometric variables absorbed 45.3% of the whole shape variation. PC1 (16.50% of total variance) ordered the specimens according to the relative position of the caudal peduncle (landmarks 4–8; Fig. 1A); individuals with a higher position of the caudal peduncle scored positive values of PC1, whereas fish with lower positioned peduncles had negative scores. PC2 explained a similar amount of variance (15.1%). In this case, positive scores corresponded to rather elongated fish, whereas those with deeper bodies had negative values for PC2. Finally, PC3 (13.7%) summarized variation in the relative shape of the head (landmarks 1 and 2).

The first three principal components of a second PCA on the same morphometric variables for the fish recaptured in February, after a period of five months in the river, explained 57.3% of the total variance. Again, PC1 (27.8%) ordered specimens according to peduncle relative elevation. PC2 (16.7%) was associated with body depth and head size; on the negative side of PC2 scored ‘big head’ specimens with dorsal and pelvic fins located in a more anterior position which conferred them a more robust and a less elongated appearance (Fig. 1B). In this case, PC3 (12.8%) assimilated changes in the relative positions of landmark 1 (tip of the upper jaw), eye landmarks, and the insertions of dorsal and pelvic fins. Therefore, the major components of shape remained unchanged during the study period.

As expected, we observed a highly significant, positive relationship between metabolic rate and body mass (*R*^2^ = 0.53, *F*_1,175_ = 193.65, *P* < 0.000001; log-transformed data). Centroid size was highly correlated to fish weight and length both at the beginning (weight: *R*^2^ = 0.97, *F*_1,210_ = 7940.75, *P* < 0.000001; length: *R*^2^ = 0.99, *F*_1,210_ = 17,726.54, *P* < 0.000001; see Supplementary Fig. S1 online) and at the end (weight: *R*^2^= 0.95, *F*_1,30_ = 601.57, *P* < 0.000001; length: *R*^2^ = 0.99, *F*_1,30_ = 4716.24, *P* < 0.000001; see Supplementary Fig. S2 online) of the experiment. All these correlations were significant after controlling for FDR (false discovery rate; Benjamini–Hochberg procedure) and FWER (family-wise error rate; Bonferroni sequential correction).

### Dispersal and settlement

**Figure 2:**
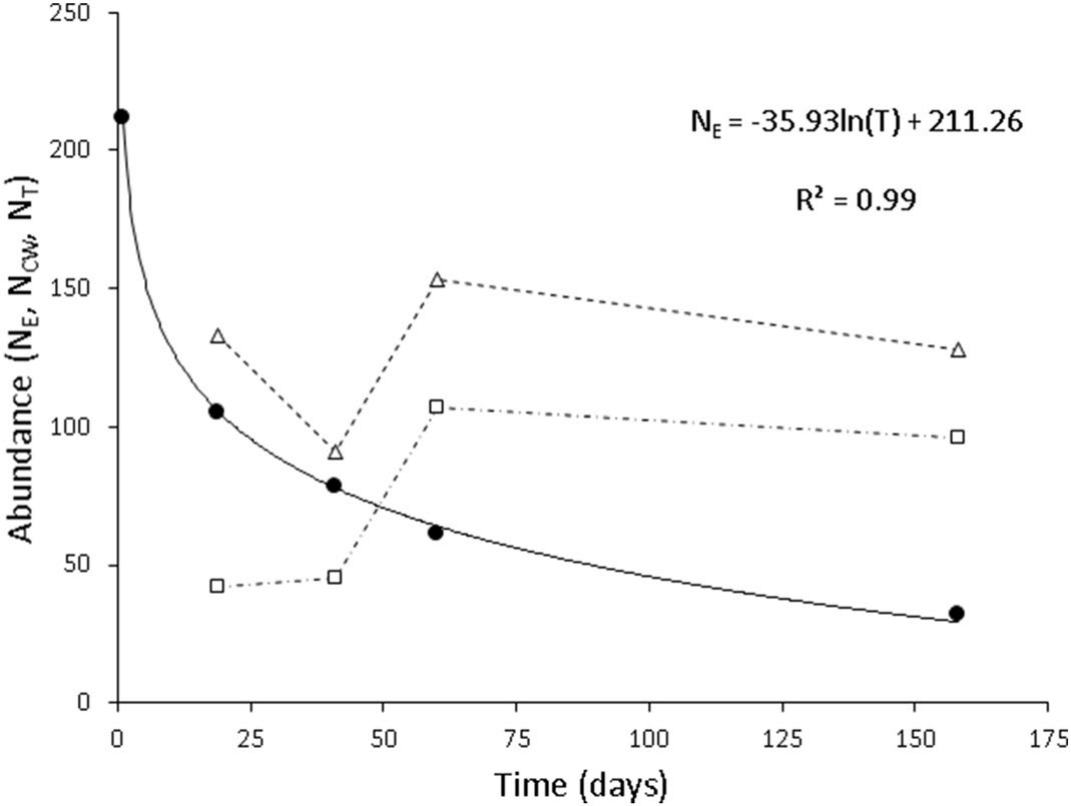
Residency decay of experimental fish (solid circles) in the study area (− 600, + 500 m from the release point). Residency was calculated as the minimum number of experimental fish present in the study section (fish captured at each sampling date or later). The solid line represents the fitting of a logarithmic model. Open squares are numbers of wild fish captured at each time, and triangles are total captures (wild and experimental fish).

**Figure 3:**
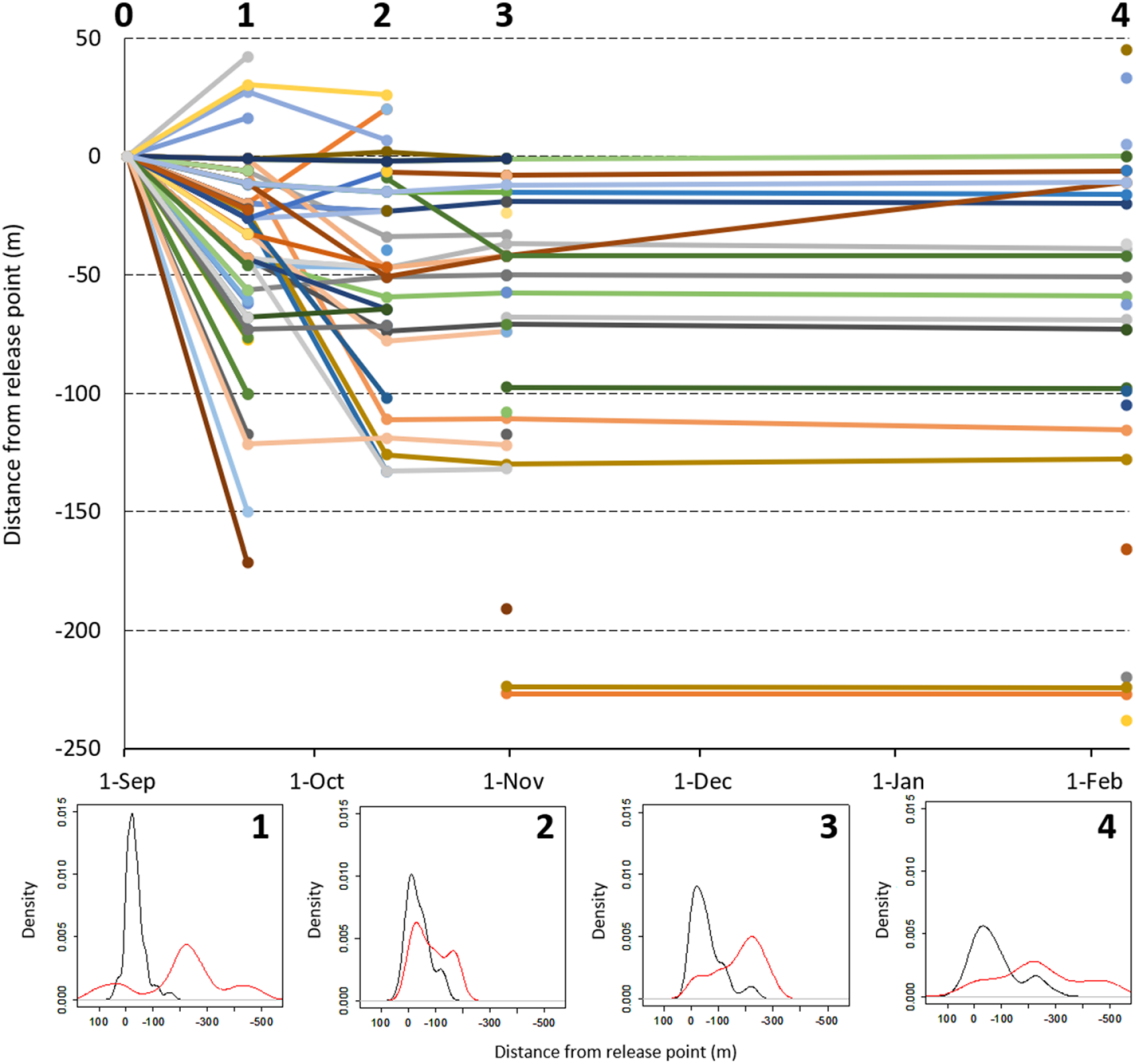
Evolution of individual dispersal distances during the experimental period (158 days). Frequency graphs (1, 2, 3 and 4) were performed by using Kernel interpolation; frequencies of experimental and wild fish are indicated by black and red lines, respectively (0: time at release; 1: 19 days later; 2: 41 days, 3: 60 days; 4: 158 days).

From 18th September to 6th February, a total of 105 fish were recaptured at least once in the Santianes River. Captures of experimental fish decreased over time, especially at the start of the experiment (Table 1). This was due, at least partially, to a logarithmic decay of the number of experimental fish (*F*_1,3_ = 3249.60, *P* = 0.000012; Fig. 2; Table 1). However, the numbers of wild fish increased between days 41 and 60; as a result, trout abundance (wild and experimental) tended to keep steady over the study period (Fig. 3).

**Table 1:** Clark–Evans index of aggregation and variance-to-mean ratio (VMR index) for the four sampling events (19, 41, 60, and 158 days after release).

Time (days)	Experimental fish						Experimental and wild fish				
	*N*_E_	*N*_Ce_	Clark & Evans		VMR		*N*_*T*_* (N*_*Cw*_)	Clark & Evans		VMR	
			R	*P*	D	*P*		R	*P*	D	*P*
0	212										
19	105	91	0.846	< 0.001	10.580	< 0.001	133 (42)	0.556	< 0.001	7.252	< 0.001
41	78	46	1.171	0.018	2.253	0.003	91 (45)	0.677	< 0.001	3.408	< 0.001
60	61	46	1.197	0.004	4.000	< 0.001	53 (107)	0.548	< 0.001	2.824	< 0.001
158	32	32	2.747	0.330	1.091	0.330	128 (96)	0.983	< 0.001	1.273	0.135

Analyses were conducted twice, for samples including or excluding the spatial positions occupied by wild fish. *N*_E_, minimum number of experimental fish in the study section at each sampling session (regardless of whether they were captured or non-captured); *N*_Ce_, *N*_*T*_*,* captures of experimental fish and total capture, respectively (number of wild fish (*N*_Cw_) in parentheses).

Most fish tended to disperse downstream, but a small fraction moved upstream; movements ranged between 2 and 289 m (Fig. 3). Dispersal activity was highest after the release; by day 19, experimental fish were captured over a river length of 214 m, from 42 m upstream to 172 m downstream the release point (+ 42, − 172). We did not find evidence of further dispersal by day 41 (+ 27, − 133), but then recorded an extension of the colonized area downstream by day 60 (− 1, − 227). In the last sampling, there was a new increase downstream but not in upstream dispersal (+ 45, − 289). Experimental fish were never detected above 45 m upstream or below 289 m downstream the release point.

Our results indicated that a large fraction of fish tended to establish territories relatively soon and then maintain these positions (Fig. 3). We verified that at least 105 and 65 of the 212 released trout were alive and stayed in the study area after 19 and 41 days, respectively. A total of 39 out of the 65 fish that could be monitored at 60 and 158 d were settled in their final location by day 19 (RS1 = 1, n = 39). Most of the fish monitored for RS2 remained in these locations (46 out of 48), as evidenced by the tight relationships between the positions recorded at days 41 and 60 (*R*^2^ = 0.97; *F*_1,21_ = 615.45, *P* < 0.00001), 41 and 158 (*R*^2^ = 0.94; *F*_1,15_ = 217.20, *P* < 0.00001), and 60 and 158 (*R*^2^ = 0.99; *F*_1,15_ = 1199.13, *P* < 0.00001; see Supplementary Fig. S3 online). Therefore, we restricted the analyses to initial survival/permanence (SURV1) and RS1. Although, in average, survivors/stayers were slightly bigger, heavier and more robust than non-recaptured fish, a probit model did not reveal significant influences associated to centroid size (*z* = 1.01, *P* = 0.314), shape (PC2; *z* = 0.99, *P* = 0.321), mass (*z* = − 0.56, *P* = 0.575) or mass-specific metabolic rate (*z* = 0.92, *P* = 0.358).

The probability to settle in the first weeks after release (RS1) was affected by shape and body mass; early settlers were heavier (*z* = − 2.10, *P* = 0.035) and had deeper bodies (*z* = − 2.40, *P* = 0.0163) than movers, but RS1 was not affected by mass-specific metabolic rate (*z* = 0.97, *P* = 0.3315). Centroid size has a marginally nonsignificant effect on RS1 (*z* = 1.90, *P* = 0.057). Additionally, we found a clear link between the timing of settlement and dispersal distances; rapid settlers moved shorter distances than late settlers (maximum dispersal distance, mean ± SE; late: 100.6 ± 11.1 m, *n* = 26; early: 41.2 ± 19.1 m, n = 39; *F*_1,63_ = 17.21, *P* = 0.0001). Fish location along the stream axis was also affected by propensity to settle. Early settlers tended to settle upper positions (either upstream or downstream but close to the releasing point) whereas late settlers moved to lower reaches; this effect was significant at the second (41 day; *P* = 0.0167), third (60 day; *P* = 0.0096) and final (158 day; *P* = 0.0074) controls, but not at the first one (19 day; *P* = 0.159). Moreover, final positions after 158 d were predicted by positions at 60 d (*F*_1,15_ = 1193.13, *P* < 0.000001) and 41 d (*F*_1,15_ = 217.20, *P* < 0.000001) but not for position 19 d after release (*F*_1,25_ = 1.20, *P* = 0.285). The statistical significance of these results was validated by both the FDR controlling procedure of Benjamini–Hochberg and the Bonferroni sequential correction to control for FWER.

Maximum dispersal distance was related to proximity to other conspecifics, but the pattern changed over time. At the start of the experiment we found a significant, positive relationship between maximum dispersal and distance to the nearest juvenile conspecific (19 day; *F*_1,89_ = 5.70, *P* = 0.02). This relationship was evident regardless of the origin of the nearest neighbor (wild trout: *F*_1,89_ = 4.81, *P* = 0.031; hatchery released trout: *F*_1,89_ = 8.89, *P* = 0.0037) and remained significant after controlling for FDR and FWER. After one month in the river, proximity to wild fish did not explain variation in maximum dispersal distance of the experimental fish (41 day: *F*_1,44_ = 0.07, *P* = 0.786; 60 day: *F*_1,44_ = 1.70, *P* = 0.199; 158 day: *F*_1,30_ = 0.11, *P* = 0.780), but the relationships was clear for the experimental fish (41 day: *F*_1,44_ = 11.87, *P* = 0.00126; 60 day: *F*_1,44_ = 14.89, *P* = 0.00037; 158 day: *F*_1,30_ = 14.22, *P* = 0.00368; validated by using the Benjamini–Hochberg procedure and Bonferroni sequential correction), thus confirming that spacing among hatchery-released fish tended to increase as they moved away from the release point.

### Spatial distribution

Exponential and gamma distributions provided the best fitting to the distribution of frequencies of dispersal distances (see Supplementary Table S1 online). After the release, spatial distribution of introduced fish showed a nonrandom pattern of dispersion that persisted for at least two months. Kernel analyses showed an initial oversaturation around the release point that decreases with distance from the release point (Fig. 3); this pattern softened over time. Besides, the frequency distributions of all the captured individuals indicated a bimodal distribution that reflects the spatial segregation between wild and experimental fish (Fig. 3). A clear increase of aggregation values and leptokurtosis was observed in the second sampling (41 day), seemingly due to upstream movements of wild fish (Fig. 3). Apparently, wild fish tended to re-occupy the upper sections which increased the spatial overlapping of wild and introduced fish (Fig. 3). Thereafter, wild fish seemingly spread downstream (Fig. 3), but this paralleled an increase in the number of wild fish by day 60. In contrast, frequency distributions for experimental fish showed a progressive reduction of leptokurtosis and an increment of mean Euclidean distances to the nearest neighbour (Table 1). As a result, we observed a consistent reduction in the aggregation of the experimental fish as they disperse from the releasing point (Table 1). However, when the joint distribution of wild and experimental fish is considered, the Clark-Evans index indicated patterns significantly more clustered than random over the entire study period. The VMR index of dispersion revealed a highly clustered distribution by day 19, then a lower but still significant aggregation tendency after 41 and 60 days, and a random distribution after 158 days, regardless of whether wild fish were considered or not in the calculations (Table 1). It should be noted that these indexes can reflect different patterns in relation to the effective scale or sampling area, and therefore they can provide complementary information.

### Size, shape, and metabolic rate as dispersal predictors

**Figure 4:**
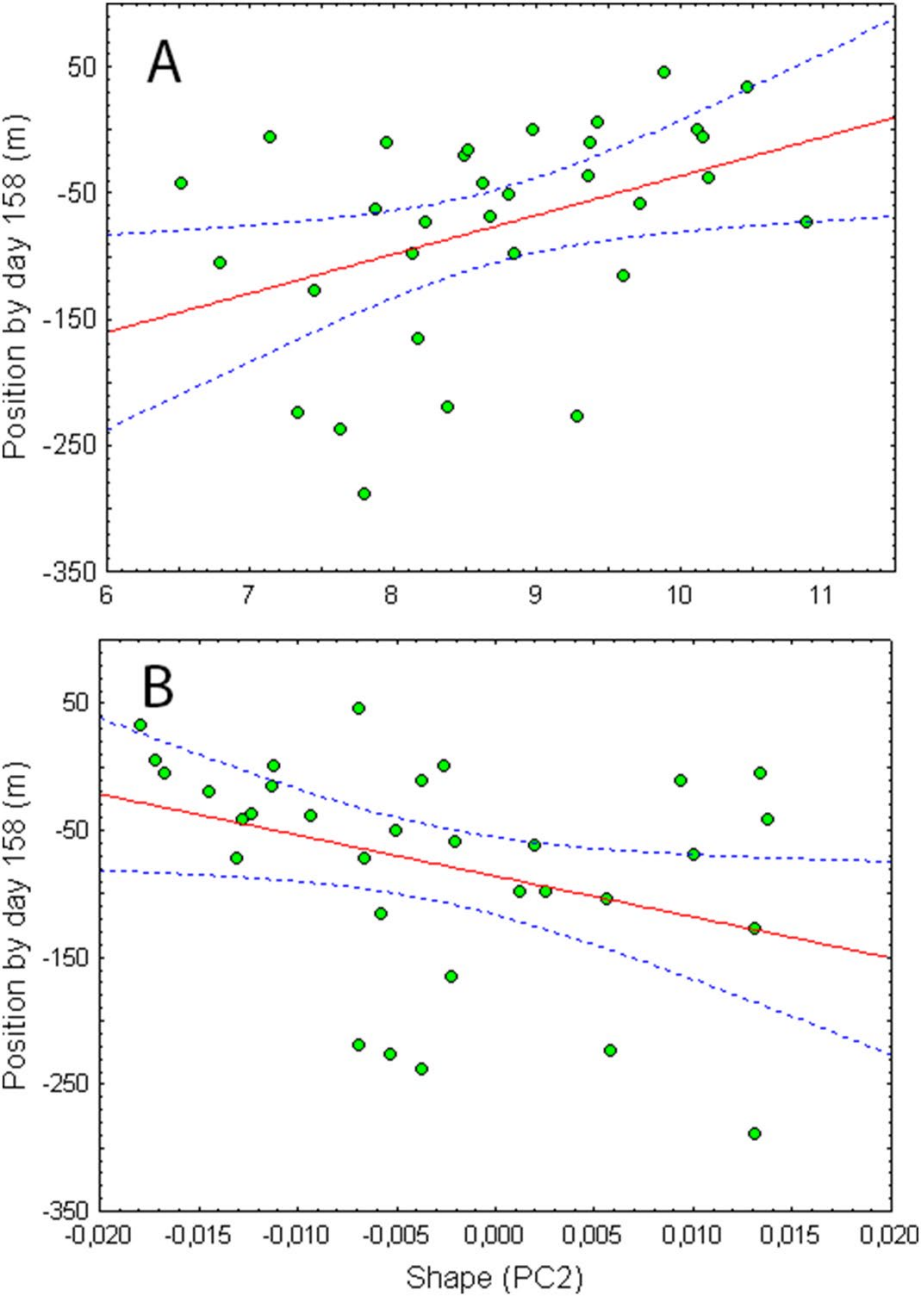
Regression of settlement positions by day 158 (distances from the release point at the end of the experiment) on (**A**) initial size (centroid size before release; *F*_1,30_ = 5.71, *P* = 0.028) and (**B**) shape before release (PC2; *F*_1,30_ = 4.56, *P* = 0.041). Negative and positive values for ‘position’ refer to locations downstream and upstream of the release point, respectively.

Over the study period, the fish recaptured at the end of the experiment showed significant growth in mass (mean ± 1SD; August: 3.94 ± 1.63 g; February: 6.69 ± 2.98 g; repeated-measures ANOVA: *F*_1,31_ = 149.43, *P* < 0.00001) and length (August: 74.73 ± 9.05 mm; February: 84.62 ± 11.99 mm; repeated-measures ANOVA: *F*_1,31_ = 141.22, *P* < 0.00001). At the end of the monitoring period, experimental and wild fish did not differ in length (*F*_1,53_ = 0.24, *P* = 0.62) or mass (*F*_1,53_ = 1.31, *P* = 0.26). When considering only traits measured at the start of the experiment, the GLM models that best explained settlement position were based on centroid size and shape (PC2) (Table 2). Most movements were downstream, and smaller and more elongated fish attained lower positions, whereas the largest and most robust individuals tended to move upstream and remained close to the release point (Fig. 4). For dispersal distances (maximum distance from the release point), the best models involved shape (PC2) and metabolic rate (Table 2); fish with deeper bodies and higher metabolic rates moved shorter distances than elongated, low-SMR fish.

**Table 2:** Comparison of AIC_C_ deltas and weights for general linear models testing the effects of metabolic rate (SMR), initial body size (centroid size, CS) and initial shape (PC1, PC2) on secondary dispersal (maximum distance from the release point) and settlement positions of juvenile brown trout along the stream axis after day 60.

Response	Candidate model	K	R^2^	AIC_C_	ΔAIC_C_	w AIC_C_	ER
Position (N = 66)	SMR + PC1 + PC2 + PC3 + CS	7	0.0268	751.72	9.16	0.0067	97.71
	PC1 + PC2 + PC3 + CS	6		749.24	6.69	0.0233	28.31
	PC1 + PC2 + CS	5		746.85	4.29	0.0770	8.55
	**PC2 + CS**	4		744.63	**2.07**	**0.2342**	**2.81**
	**CS**	3		742.56	**0.00**	**0.6588**	**1.00**
Distance (N = 66)	SMR + PC1 + PC2 + PC3 + CS	7	0.0261	747.63	9.08	0.0069	93.88
	SMR + PC1 + PC2 + PC3	6		745.12	6.58	0.0243	26.80
	SMR + PC1 + PC2	5		742.76	4.21	0.0792	8.22
	**SMR + PC2**	4		740.56	**2.01**	**0.2385**	**2.73**
	**PC2**	3		738.55	**0.00**	**0.6511**	**1.00**

Shown are the number of parameters (K), the resulting AIC_C_, the ΔAIC_C_, AIC_C_ weight and the evidence ratios (ER). The best model and models roughly equivalent to the best (ΔAIC_C_ ≈ 2 or lower) are highlighted in bold.

For the second data set (measures recorded at the beginning and at the end of the experiment) shape emerged as the most informative factor; the best model for fish position after 158 days included both initial and final PC scores for the robustness-elongation axis (PC2_AUG_ + PC2_FEB_) but a model involving peduncle position at the end of the experiment (PC1_FEB_) was equally plausible (Table 3). We obtained similar results for dispersal distances, but in this case only the robustness-elongation axis contributed to the most plausible models (Table 3).

**Table 3:** Comparison of AIC_C_ deltas and weights for general linear models testing the effects of metabolic rate (SMR; measured before release), initial body size (centroid size, CS_aug_), final size (CS_feb_), initial shape (PC1_aug_, PC2_aug_), and final shape (PC1_feb_, PC2_feb_) on secondary dispersal (maximum distance from the release point) and settlement positions of juvenile brown trout along the stream axis at the end of the experiment (day 158).

Response	Candidate model	K	R^2^	AIC_C_	ΔAIC_C_	w AIC_C_	ER
Position (N = 26)	PC1_aug_ + PC2_aug_ + CS_aug_ + PC1_feb_ + PC2_feb_ + CS_feb_ + SMR	9	0.2942	323.89	17.46	0.0001	6171.02
	PC1_aug_ + PC2_aug_ + CS_aug_ + PC1_feb_ + PC2_feb_ + SMR	8		319.11	12.68	0.0012	565.61
	PC2_aug_ + CS_aug_ + PC1_feb_ + PC2_feb_ + SMR	7		314.87	8.44	0.0098	67.95
	PC2_aug_ + CS_aug_ + PC1_feb_ + PC2_feb_	6		311.27	4.84	0.0591	11.23
	**PC2**_**aug**_** + PC1**_**feb**_** + PC2**_**feb**_	5		308.26	**1.83**	**0.2664**	**2.49**
	**PC2**_**aug**_** + PC2**_**feb**_	4		306.43	**0.00**	**0.6635**	**1.00**
Distance (N = 26)	PC1_aug_ + PC2_aug_ + CS_aug_ + PC1_feb_ + PC2_feb_ + CS_feb_ + SMR	9	0.2762	321.10	18.64	0.0001	11,132.44
	PC1_aug_ + PC2_aug_ + PC1_feb_ + PC2_feb_ + CS_feb_ + SMR	8		316.33	13.87	0.0007	1025.48
	PC1_aug_ + PC2_aug_ + PC1_feb_ + PC2_feb_ + CS_feb_	7		312.15	9.69	0.0060	126.94
	PC2_aug_ + PC1_feb_ + PC2_feb_ + CS_feb_	6		308.44	5.98	0.0384	19.85
	PC2_aug_ + PC1_feb_ + PC2_feb_	5		305.21	2.75	0.1931	3.95
	**PC2**_**aug**_** + PC2**_**feb**_	4		302.46	**0.00**	0.7618	**1.00**

Shown are the number of parameters (K), the resulting AIC_C_, the ΔAIC_C_, AIC_C_ weight and the evidence ratios (ER). The best model and models roughly equivalent to the best (ΔAIC_C_ ≈ 2 or lower) are highlighted in bold.

*Six out of the 32 fish captured by day 158 had not been measured for SMR and therefore were excluded from these analyses.

## Discussion

This study showed that individual size and body shape can be used as reliable predictors of settlement and dispersal for artificially-reared fish when introduced in natural environments. Large, deep-bodied individuals with thicker caudal peduncles and higher metabolic rates (absolute, size-dependent SMR) tended to settle in the upper sections and fixed position relatively soon and close to the point of release. In contrast, smaller and more elongated fish were prone to disperse downstream and larger distances than bigger and more robust trout. Furthermore, these smaller and more elongated fish showed a higher propensity to delay settlement. To our knowledge, this is the first demonstration of a direct link between fish shape and secondary dispersal and spatial spreading patterns.

Our results were consistent with previous research suggesting that (1) both translocated and wild juvenile salmonids are prone to move downstream, and (2) smaller fish tended to move longer distances than larger fish^4,25^. Moreover, dispersal patterns can be affected by sex. For instance, in very young (fry) masu salmon (*Oncorhynchus masou*), males tend to disperse upstream or stay near the release site, while females tend to move downstream^26^; this difference between males and females occurs soon after the emergence from gravels and seems to be determined genetically^26^. Our study did not seek to distinguish between males and females and, therefore, we cannot discard that the observed links between fish size or shape and dispersal could be also associated with sex. Sex-biased dispersal is common among vertebrates^27^, and male-biased (but not female-biased) dispersal has been reported for brook trout (*Salvelinus fontinalis*)^28^ and brown trout^29^. Although, to our knowledge, there is no evidence for sexual dimorphism in size and shape in these earlier stages of salmonid fishes, the potential connection between the sex of juvenile fish and dispersal propensity deserved further research.

Multimodel inference suggested also that fish with higher metabolic rates, which might confer them superior competitive abilities^30,31^, settled earlier and disperse lower distances (but see^32,33^). However, because the tight association between SMR and body size, this effect must be interpreted with caution; in fact, size-adjusted standard metabolic rates did not contribute substantially to predict dispersal distances and final positions along the stream channel. Thus, we failed to identify metabolic rate per se as a plausible predictor of dispersal and exploratory behavior^31^, and more research will be required to elucidate this particular point.

There are evidences that unintended selection in hatchery environments can promote boldness, and that bold individuals are more likely to become dominant than their shyer conspecifics^34^. Moreover, since boldness is a potential driver for exploratory behavior, bolder, more aggressive individuals should be prone for exploring new areas^7,24^ and disperse larger distances than subordinated conspecifics^24^ (i.e., the ‘explorer’ hypothesis). Our results, though yield mixed support for this scenario, do not support the explorer hypothesis in the brown trout. Presumably, because both a large size and a deep-bodied shape confer a higher burst speed, bigger and more robust fish are more likely to be the winners in competitive interactions, regardless of these are related to exploitative or interference competition. If so, the observed link between size and shape and dispersion patterns would support the defender hypothesis: more competitive fish acquire territories quickly and close to the release point, and then hold these positions^14^, which must involve some ability to outcompete both released and resident wild fish.

Settlement is a final and determinant stage of dispersal^35^. Our results indicated that a large proportion of the fish settled on their final locations within a relatively short period (60.0% settlement before 19 days, and 95.8% settlement before 41 days) and therefore dispersal distance at 41-days was a good proxy for final dispersal. One remarkable result was that rapid settlers moved only short distances. This implies that they were able to find suitable habitat patches, and to gain access to territories after competition with conspecifics if these were occupied by either wild or hatchery-reared fish. On the other hand, the fish that moved the greatest distances were also the latest to settle. This suggests that smaller and more elongated fish were likely subordinate and had a low competitive ability, and therefore they could be recurrently displaced in their downwards drift^14^. This would suggest a trade-off between leaving a suitable site and searching for a better territory, where fish are prone to keep on a conservative strategy^36^. Seemingly, there is a tendency to avoid the risk associated with searching for a better place (e.g., risk of entering poorer patches, or patches with an intense competition or a higher risk of predation). Paradoxically, despite bigger, deep-bodied, and more aggressive fish would take lower risks than smaller subordinate ones, the former stayed and the later moved. Again, this upholds the ‘defender’ hypothesis but not the ‘explorer’ hypothesis.

The high consistency of fish positions over the study period support previous work on brown trout^36^ and is compatible with the ‘restricted-movement paradigm’^37,38^. We are aware that this concept involves spatial and temporal scales wider than those used here, and that such constancy can be broken as fish grow and energetic requirements change, but the tendency to remain in a restricted area over short-term scales suggest also the existence of some inherent sedentary behaviour.

We observed an unambiguous relation between body shape and dispersal. The relevant shape dimension was related to body and head depths and thickness of the caudal peduncle. The general pattern of shape variation, summarized by the first two axes extracted from the PCAs, remained unchanged during the experiment. Deep-bodied fish with thick peduncles tended to settle very close to the release point (with extreme deep-bodied shapes moving upstream), whereas fish with highly elongated bodies and thin peduncles tended to settle away from the release point and moved always downstream. Even so, for PC2 there was a trend for a transition from elongated to robust shapes^39^; this shift could be provoked by the burst swimming or intermittent (burst-and-coast) swimming styles favoured in the riverine environment, while constant currents in the hatchery tanks would favour sustained swimming^39^. Although body shape has a direct influence on swimming performance^40,41^, understanding how fish shape influences dispersal is not straightforward, and likely it depends of spatial scale (i.e., long- vs short-distance dispersal^42^). More elongated shapes favouring endurance can be advantageous for movements over long distances and this clearly could favour long-distance active dispersal; this is the case of pre-migratory forms of anadromous salmonids^43,44^. In contrast, deep-bodied shapes associated with burst swimming are more likely to play a role in prey-predator and competitive interactions^45^. Robust shapes with a bigger dorsoventral musculature are linked to explosive swimming^40,46^, which is essential in aggressive movements and can promote social dominance in a context of social interactions. In our study system, where dispersal occurs over a small spatial scale and mainly downstream, the continuum robust/elongated shape factor should not be determinant of dispersal distances per se (via swimming performance), and the relation between shape and dominance seems the most plausible explanation for the observed linkage between shape and distance^14,19^.

The introduced fish might have displaced the local fish at an early moment, but later on they showed lower survival or an inferior ability to remain in the study sections than wild fish. This is consistent with previous research showing that prior residence conferred a competitive advantage to juvenile brown trout^47,48^, and that recently introduced fish moved more than residents of similar size regardless of they were wild or hatchery fish^36^. Additionally, our results indicated that spatial overlapping between wild and introduced fish occurred predominantly in the lower reaches of the study section, suggesting that wild fish could be able to recover some of the area occupied previously by smaller, more elongated fish, but not by those that grew faster and shifted earlier to more robust shapes, which in turn settled closer to the release point. This is supported by previous work demonstrating that stocking of hatchery-raised trout can have a negative impact on resident trout in the short-term but did not translate into demographic effects at the mid- or long-term^49^. We could not explain the apparent increase in the abundance of wild fish in the study section between days 41 and 60, but most likely it was related to a lower catchability of wild fish over the first weeks after the introduction of hatchery fish; an inferior catchability might be derived from either a shift in the behaviour or microhabitat used by wild fish, or a dilution effect associated with the high density in the weeks following stocking.

The Santianes River is a very small headwater stream that runs mostly over a siliceous bedrock, with a low productivity. In these conditions, one can expect strong competition for food and moderate to high mortality/emigration rates that can increase after stocking events^25,36,49^. In the Santianes River, the density of introduced fish showed a pronounced initial decline and then remained relatively stable, with abundance declining gradually through the autumn, a pattern of decay similar to the observed in other salmonid populations^25,49^, but not always^36^. Although our experiment did not allow to distinguish between natural mortality and emigration, thus preventing a clear interpretation, the fish that disappeared during this first pulse did not differ in size, shape, or mass-specific metabolic rate from those remaining in the study area. Therefore, which factors are involved in these early pulses^25,49^, remains an open question.

Here we showed that settlement location and secondary dispersal can be predicted by individual size and shape of the fish at least five months earlier. This identifies a suite of correlated traits (size, shape, and dispersal behaviour) that support of the pace-of-life syndrome (POLS) hypothesis, which can have important consequences for the persistence of aquatic species constrained to disperse within dendritic riverine networks with predominantly unidirectional gene flow^50^. However, neither size nor shape are reliable predictors of survival or settlement in the area of release, and therefore, in a first step, the settlement of introduced fish after release in natural environments can have a stochastic component. In fact, the results on size and shape clearly supported the defender hypothesis (more aggressive and dominant individuals will settle earlier and closer to the release or emergence area^14^). This can have sound implications for the enhancement of populations based on supplementation and stocking^7^. If the defender strategy is widespread, the introduced animals might have either a negative effect on wild individuals or a low expectancy of settlement depending on the relative expression of specific traits in introduced and resident animals^36,49^.

The observation that morphological and physiological traits can be used to predict potential secondary dispersal uncovers important implications for fine scale stock management and, particularly, population reinforcement through targeted stocking actions. Moreover, discrete grading based on differences in shape and size is feasible at low costs by using simple measurements and even, with previous training, directly by eye. This allows the selection of different phenotypes targeted at specific purposes, or conducting precise stocking actions at a fine spatial scale to adjust the introduction of new individuals to the characteristics of the recipient population and reduce the potential of negative impacts on the native fish^25,36,49^. For instance, selective stocking in reaches with contrasting densities of resident fish can require ‘rapid’ settlers for strong stocking of overexploited reaches, but ‘late’ settlers to fill potential gaps in reaches maintaining higher densities of autochthonous fish.

In summary, future research should explore quantitatively the interactive effects of context (e.g. densities of resident and introduced populations) and key traits (e.g., size, age, metabolic rates, and shapes of introduced and resident animals) to optimize stocking actions aimed at the population reinforcement. Finally, this study is a good example that combining physiological, behavioural, and morphometric data with longitudinal studies under natural conditions can be a successful approach to unravel the complex interactions between environmental and genetic factors affecting dispersal.

## Methods

### Experimental fish

The experimental fish were the offspring of wild trout caught in the Sella River (Asturias, northern Spain) by the staff of the Service of Natural Environment of the Principality of Asturias. The brown trout from Sella River exhibit partial anadromy, although the parental fish used for this experiment were nonanadromous individuals. A total of 30 females and 30 males were used in multiple crosses to generate a pool of several thousands of fertilised eggs, which captures a representative fraction of the genetic variation in the source population. In each cross, the eggs from 3–4 females were pooled and then fertilised with the sperm of 3–4 males. Embryos and juveniles were reared at the facilities of the Espinareu River hatchery centre (Centro Ictiogénico de Infiesto, Principado de Asturias). After three months, a random sample of these age 0 + fish (N ≈ 300) was transferred to the facilities of University of Oviedo and stocked in circular holding 80-L PE tanks with an input flow of 12 L min^−1^ at a density of 40 fish per tank. The tanks were equipped with a mesh cylinder containing a drainage tube in the middle to set water level and prevent the fish from entering the drain. Tanks were fed with tap water from Oviedo municipality previously treated to remove chlorine and chloramine. We fed fish twice a day up to they were satiated (maintenance rations). In doing so, we prevented significant changes in juvenile mass over the 3-wks period during which we carried out the measurement of metabolic rate (ANOVA, *P* = 0.68). We used a natural photoperiod and temperature was set at 14 ± 2 °C.

### Estimation of standard metabolic rates

Standard metabolic rate (SMR) was measured by flow-through respirometry^16,51,52^ with an oxygen meter (Strathkelvin Instruments Ltd., Model 782, Glasgow, Scotland) connected to a microcathode oxygen electrode (Strathkelvin Instruments, Model 1302) placed inside a thermostatted cell (Strathkelvin Instruments, MC 100). The system consisted of 19 cylindrical chambers (18 test plus 1 control chambers, 30 mm × 200 mm, internal volume 81.7 cm^3^) immersed in water at a constant temperature of 14.82 ± 0.16 °C. All the 19 chambers were independently supplied in parallel by saturated-in-oxygen water.

Prior to measuring SMR, the fish were acclimatised for at least four weeks at the test temperature (14 °C). Oxygen consumption measurements were conducted on 12 days between 10 and 30 August, and from 10:00 to 16:00 h to reduce potential circadian effects. Each day, we selected a random sample of 14–18 fish and fasted them for 48-h to arrest d food digestion. Then, the fish were placed into the respirometry chambers for additional 15 h in darkness to ensure that rates of oxygen consumption get into a steady basal state.

We measured oxygen concentration from inflow and outflow water samples at each chamber. For each fish, SMR was recorded at least twice, with a time interval of 90 min between each measure, in order to ensure that conditions within chambers had reached initial state^16,52^. SMRs were derived from the difference between inflow and outflow oxygen content^51,52^. We adjusted flow rate (mean ± 1SD: 15.71 ± 1.39 ml min^−1^) and trial duration to ensure that consumption rates were not affected by low O_2_ levels. All valid measurements had values of O_2_ depletion lower than 10% (final oxygen saturation greater than 90%). We obtained estimates of SMR for a total of 177 fish.

### Image acquisition and shape analysis

Digital images were taken immediately after the respirometry trials. We used a standardised protocol to acquire the digital images for morphometric analyses. Prior to photographing and tagging, fish were anesthetized using benzocaine (Ethyl 4-aminobenzoate; Sigma-Aldrich, Darmstadt, Germany. Product number: E1501. Ref.: 112909; final concentration: 5 mg L^−1^). To avoid potential arching effects^53^, they were aligned on their right side in a relaxed position using midline as reference to linearity. All images presenting some indication of curvature were discarded. Then the fish were weighed to the nearest 0.01 g, and individually tagged with colour VIE codes (Visible Implant Elastomers, NorthWest Marine Tech, WA, USA)^54^. Each tag consisted of 1–3 mm stripes of green, orange, pink, or blue elastomer injected below transparent skin with an insulin syringe mounted with a 29-gauge needle. These stripes were inserted in one or more of 11 locations (fins: left/right pectoral, left/right pelvic, anal, upper/medium/lower caudal, and dorsal; postocular adipose eyelid tissue: left/right). In doing so, we generated 247 individual codes by using unique combinations of colour (green, orange, pink, blue) and tag locations; most of the fish were tagged by using stripes of VIE in 1–3 locations, and only in a few cases we used 4 (n = 7) or 5 (n = 1) strips of VIE.

**Figure 5:**
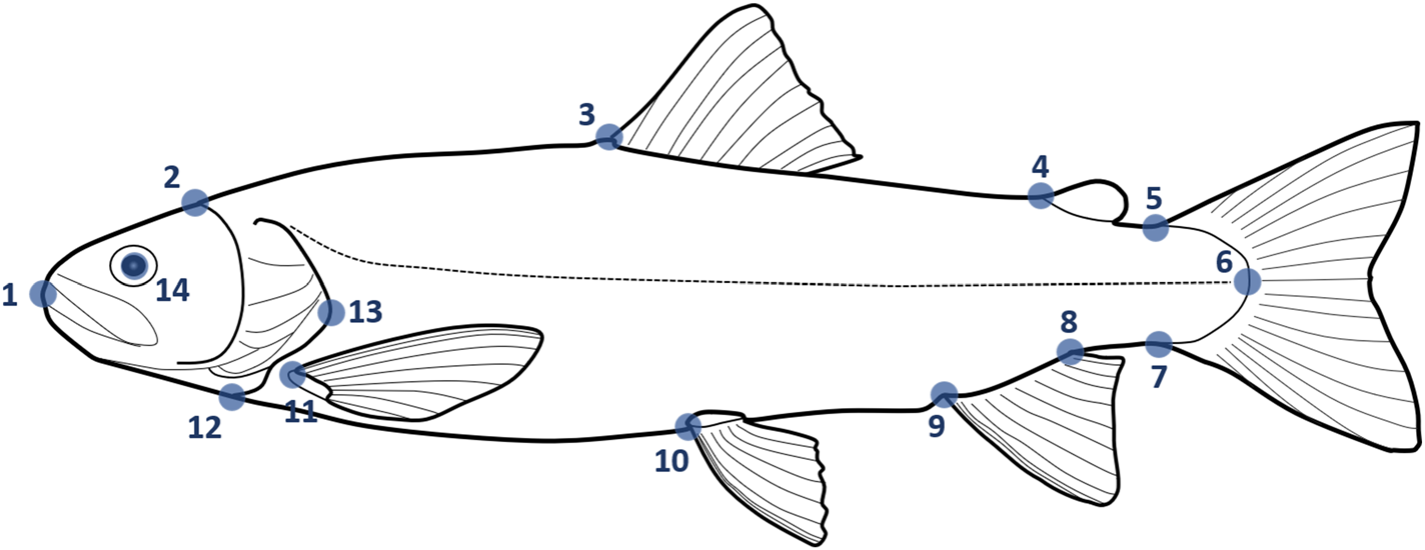
Collection of landmarks used for the morphometric analyses of brown trout shape: (1) tip of upper jaw; (2) posterior supraoccipital notch, (3) anterior insertion of dorsal fin; (4) origin of adipose fin; (5 & 7) anterior junction of dorsal and ventral membrane from caudal fin; (6) intersection of lateral line and membrane of caudal fin; (8 & 9) posterior and anterior insertion of anal; (10) origin of pelvic fin; (11) origin of pectoral fin; (12) ventral insertion between operculum and the body outline; (13) posterior tip of operculum; (14) centre of orbital. Drawing credit: Jorge-Rubén Sánchez-González.

We generated a two-dimensional landmarks system on digitized pictures using tpsDig2 v.2.17^55^ and obtained a set of 14 homologous landmarks (Fig. 5). Landmarks were superimposed, scaled, aligned and rotated to a consensus shape using Thin-Plate Spline (TPS) analysis^56^ by using tpsRelw version 1.53^57^. This generated 24 partial warps (22 uniform and 2 non uniform scores).

To examine the potential relationships between fish shape, dispersal, and metabolic rate, we conducted two morphometric (TPS) analyses: the first one put the focus on shape before the fish were released in the river (images taken at the end of the respirometry trials; 10–30 August), whereas the second pointed out to shape variation at the end of the study period (158 days later; images taken at the last sampling operation). By doing so, implications of the initial and final shape on fish dispersal could be analysed. Centroid sizes (the square root of the summed square distances of each landmark from the centroid of the landmark configuration^56^) were computed using tpsRelw version 1.53 and used as a proxy of body size.

### Monitoring dispersal

**Figure 6:**
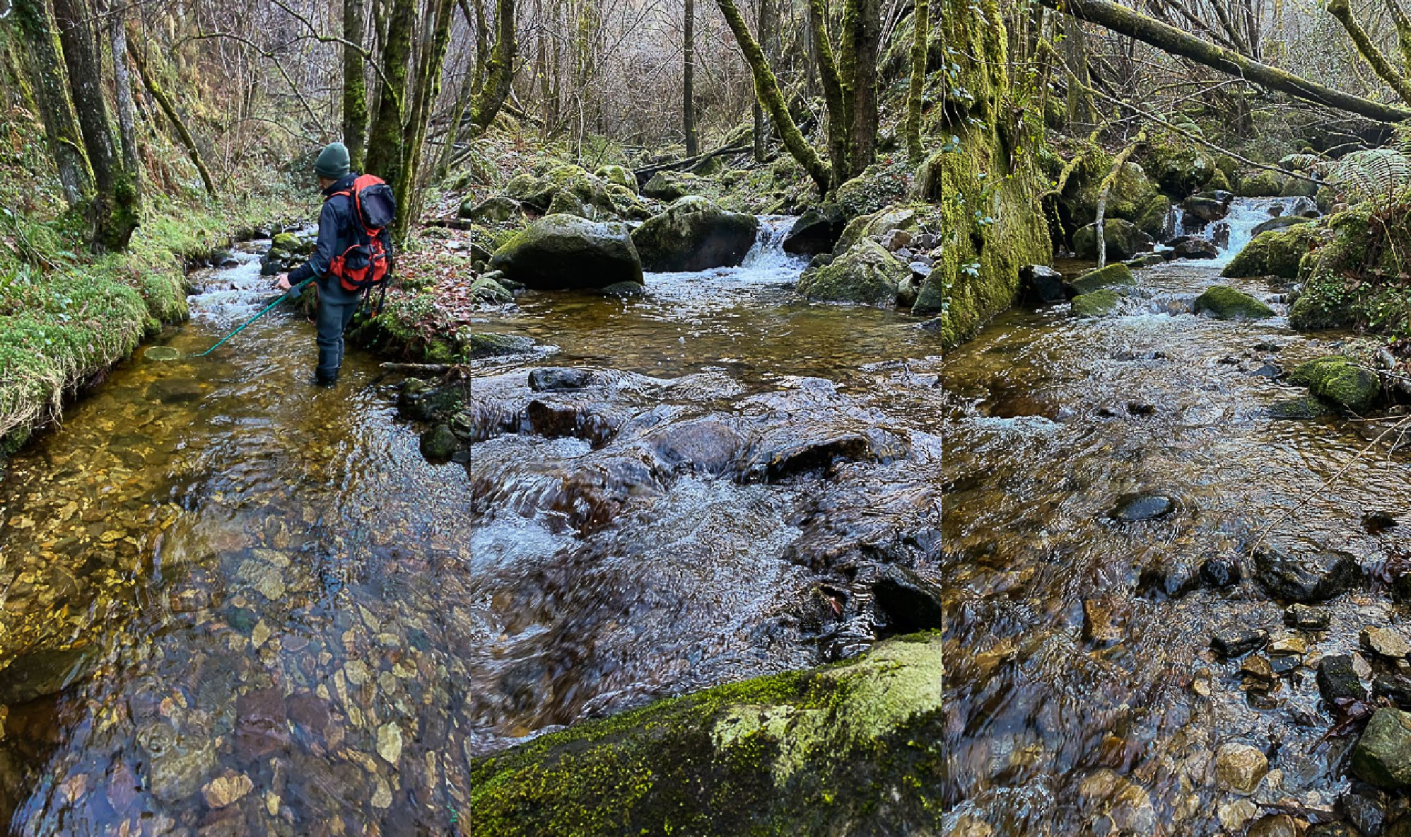
Examples of the experimental sections in Santianes River. The photographs were taken by mid-January and represent typical flow conditions during autumn and winter. Photo credit: Alfredo G. Nicieza.

The release experiment was conducted in the Santianes River (43.41984° N, 5.04075° W; 20–400 m.a.s.l.), a small headwater, coastal stream (Strahler stream order 1^58^) tributary of river Sella (Asturias, northwest Spain); therefore it can be considered as a part of the same system that the source of the experimental fish. The stream channel has a length of 3.75 km, and channel width ranges from 0.9 m in the upper sections to 3.8 m in the lower reaches. Stream structure is highly heterogeneous, and includes a succession of pools, riffles, runs, and small waterfalls (Fig. 6). Baseflow is around 0.07–0.1 m^3^ s^−1^. Topographic shadows and well-developed riparian forest prevent direct solar radiation during most time, and therefore water temperatures show very little variation both daily and seasonally (usually above 7 °C in winter and below 18 °C during summer). Canopy cover is near 100% in most of the channel river, and there is abundant debris that increases spatial complexity at the fine scale.

On 1st September 2006, a total of 212 juvenile trout (8-mo old; mean wet weight ± SD = 3.40 ± 1.43 g) were released in a small pool (~ 2 × 2 m; 45 cm maximum depth). Of these, 177 individuals were screened for SMR and shape, and 35 only for morphometric data. We established a study section along a reach of 1210 m (500 m upstream and 710 m downstream the releasing point). Within this section, channel width is on average 2 m (mean ± SD: 1.98 ± 0.68 m; range: 0.8–3.6 m), and differences in current speed or streamflow between upper and lower reaches are negligible. This reach is delimited upstream and downstream by two waterfalls (≈ 10 m) that block upstream movements. The study reach contains a small population of brown trout (hereafter, wild fish), but large predatory trout (> 210 mm) were absent and no other fish species were present; the abundance of adult trout in the study section was very low (< 0.04 ind m^−2^). European eel (*Anguilla anguilla*) and juvenile Atlantic salmon (*Salmo salar*) are present only in the lower reaches of Santianes River (outside the study area), downstream the first waterfall.

The entire study section (1210 m) was sampled four times between September and February to detect movements and changes in the locations of experimental fish. Sampling operations (19, 41, 60, and 158 day from the release date) were conducted at night because salmonid activity can be negatively correlated with light intensity, especially during autumn and winter^59–61^. We delimited small subsections of about 5–10 m length using natural elements of the river topography that limit fish escapement (e.g. rapids, small waterfalls). Then, each section was sampled twice; first, we used torchlights and hand nets to detect and extract most of the active fish with minimal displacement. Captured fish were placed into individual nets for immediate processing. For each fish, we recorded the exact position (measured as the distance to the focal releasing point), whether they were wild or experimental fish, and in the latter case, the individual identification code. Then they were kept in plastic buckets and the subsection was electrofished to capture unseen or escaped fish. After recording the data, the fish were released in the point of capture taking care they were not displaced upstream. Since the experimental fish were individually marked, we were able to discard the transference of fish between adjacent sections during fishing operations. To evaluate potential interactions between introduced and native tout, we also recorded the locations where juvenile wild fish (only age 0 +) were captured. This procedure was repeated in all the subsections of the study reach. Sampling was conducted upstream, from lower to upper reaches. In the last control (6 February, 158 days), all the experimental fish were anaesthetized with benzocaine, weighed (± 0.01 g), and photographed for further morphometric analysis.

Low intensity electrofishing was carried out with a portable back-pack equipment (BSE model EFGI 650; Bretschneider-Spezialelektronik, Chemnitz, Germany). We set parameters at 315 V or lower, 60 Hz, and 3 ms. Under these conditions, electrofishing has a minimal incidence on fish survival^62^ and risk of damage is minimised; to our knowledge, no fish were killed or injured during electrofishing operations. Sampling was always performed under optimal conditions of water flow and transparency (Fig. 6). Sampling was carried out under permits #200450700001838 and #200660700001066 from the Government of the Principality of Asturias. Experimental procedures complied with the country legal requirements on animal welfare at the time of performing the work (RD 1201/2005) and all the procedures were conducted in accordance with the guidelines of the Research Ethics Committee of the University of Oviedo. The members of the research team have approved licenses by the Service of Animal Welfare and Production of the Principality of Asturias to conduct experimental protocols with animals (license types C and D to A.G.N). This study was carried out in compliance with the ARRIVE guidelines (Animal Research: Reporting in Vivo Experiments) for how to report animal research in scientific publications (https://arriveguidelines.org/arrive-guidelines).

### Spatial analysis: dispersal and spatial distribution

Our analysis considered the dispersal distance as the distance from the focal releasing point to fish position at the time of recapture. Upstream movements were considered as positive and downstream as negative distances. This discriminates upstream and downstream movements and therefore it reflects unique positions along the stream axis. Graphs of dispersal frequencies were performed by using Kernel interpolation, and distribution of frequencies of distances were analysed by fitting to normal, lognormal, gamma and exponential distributions. To check whether the spatial distribution of introduced fish fits well with a non-random pattern of dispersion, we used the Clark-Evans nearest-neighbour method^63^, a measure of clumpiness based on mean Euclidean distances to the nearest neighbour, by using the *Spatstat* R’s package^64,65^; since fish distributions in small headwater streams fit well with a linear events model, we fixed the Y-coordinate (Y = 1) and set mean stream width at 2 m. Deviations from random distribution were evaluated by Monte Carlo tests. In addition, we calculated the dispersion index or variance-to-mean ratio (VMR), a quadrat-based method. To calculate VMRs we used 10-m subsections, distributed along the detection area on each occasion (i.e., between the highest and the lowest positions of experimental fish). Spatial analyses were done twice; the first analysis was performed on the spatial positions of the experimental fish only, whereas the second was conducted on both, experimental and wild fish. Statistical analyses were performed using R version 4.0.2 “Taking Off Again”^66^.

### Statistical analyses

To generate an orthogonal morphospace, we conducted Principal Components Analysis (PCA) on the 24 partial warp scores (using *prcomp* function in *stats* package^66^). We used the variances-covariances matrix (alpha = 0), which confers more weight to those variables which describe global aspects of shape^56^. Two separate PCAs were conducted: before release in the stream (late August) and five months after release (early February).

To explore the effect of size (centroid size), shape, mass, and mass-corrected metabolic rate on survival and the propensity to remain in the study area, and which traits could have a greater influence on time to settlement, we generated four binary variables: two ‘survival’ variables (SURV1, SURV2) and two indicators of rapid settlement (RS1, RS2). Since we were not able to distinguish between mortality and the propensity to leave the study area, fish that were never recaptured were considered as dead or long-distance migrants moving out of the study area (SURV1 = 0; fish captured at least once were labelled as SURV1 = 1). Fish that were captured either at the first or second sampling (19 and 41 days) but not at any of the last two sampling operations (60 and 158 days) were considered late deaths or displacements (SURV2 = 0; fish captured at least in one of the last two sessions were labelled as SURV2 = 1). Similarly, we categorized as rapid settlers those individuals that did not show substantial change in position (< 10 m) in successive captures, at either 19 days (RS1 = 1) or 41 days (RS2 = 1), and late settlers otherwise (RS1 = 0, RS2 = 0). Survival and permanence in the study area were inferred from the last capture of a given fish (i.e., we assumed that a fish captured only at day 60 days was also in the study area by days 19 and 41). Mass-specific SMR was obtained from the residual from the regression of log_10_-SMR (oxygen consumed per fish per unit time) on log_10_-body mass, and the PC scores were used as shape variables. We explored the effects of these individual traits on the binary response data with a probit model implemented in the *glm* function in the *MASS* package^67^. We applied both the FDR controlling procedure of Benjamini-Hochberg^68,69^, and the Bonferroni sequential correction to control for FWER, when multiples tests were conducted.

Finally, the *glm* function in the *MASS* package^67^ was used to perform a general linear model of dispersal distances as dependent variable, and centroid size, metabolic rate, and shape components (PC1–PC3) were included as explanatory variables. We evaluated separately models based on traits recorded at the beginning of the experiment and models combining initial and final traits. By doing so we can infer on the contribution of initial and final shape or size on the dispersal process. We compared the performance of these models using the second-order Akaike Information Criterion (AIC_C_). To assess the relative strengths of each candidate model we used ΔAIC_C_ and calculated AIC_C_ weights and evidence ratios (ER)^70,71^. Models with Δ_i_ values greater than 10 were considered uninformative, and those with Δ_i_ values less than 2 were assumed to be equivalent to the best model^70–73^.

## Supplementary Information

Supplementary Information.

## Data Availability

Data and R code are available from 10.6084/m9.figshare.14233979.
