# Supplementary material for: Individual differences in dominance-related traits drive dispersal and settlement in hatchery-reared juvenile brown trout
Source: Sci Rep. 2021 Mar 31;11:7277. doi: 10.1038/s41598-021-86613-4 (PMC8012712; doi:10.1038/s41598-021-86613-4)
Supplement: Supplementary file 1 — Supplementary Information. [file 41598_2021_86613_MOESM1_ESM.docx]

**Supplementary materials** for *Individual differences in dominance-related traits drive dispersal and settlement in hatchery-reared juvenile brown trout*

**J.R. Sánchez-González and A.G. Nicieza**

**Individual differences in dominance-related traits drive dispersal and settlement in hatchery-reared juvenile brown trout**

**Jorge R. Sánchez-González^1,2^ and Alfredo G. Nicieza^1,3^***

^1^ Department of Biology of Organisms and Systems, University of Oviedo, E-33006 Oviedo, Spain.

^2^ Department of Animal Science - Wildlife Section, University of Lleida, E-25198 Lleida, Spain

^3^ Research Unit of Biodiversity (UMIB:UO-CSIC-PA), Campus de Mieres, E-33600 Mieres, Spain

J.R. Sánchez-González (http://orcid.org/0000-0002-6170-7523)

A.G. Nicieza (https://orcid.org/0000-0003-4062-569X) 🖂 (agnic@uniovi.es)

Table S1. Results of fitting dispersion data to known distributions (Normal, Lognormal, Gamma and Exponential). N is the number of recaptured individuals at each sampling event. Parameters 1 and 2 are: k and σ for Normal and Lognormal distribution models; α and k for Gamma distribution model; λ for the exponential distribution. For each sampling date, second-order Akaike Information Criterion (AIC_C_) was used for model selection. Most plausible models (ΔAIC_C_ < 5) are indicated in bold. wAIC_C_ and ER are weights of AIC_C_ and evidence ratios, respectively (Burnham and Anderson 2002).

| Time after release | Model | N | K | Parameter1 | Parameter2 | AIC_C_ | ΔAIC_C_ | wAIC_C_ | ER |
| --- | --- | --- | --- | --- | --- | --- | --- | --- | --- |
| 19 days | Normal | 91 | 2 | 35.19 | 32.15 | 893.99 | 61.86 | 0.00 | >1000 |
|  | Lognormal | 91 | 2 | 3.01 | 1.3 | 857.68 | 25.55 | 0.00 | >1001 |
|  | **Gamma** | 91 | 2 | 1.04 | 0.03 | 834.11 | **1.98** | **0.27** | **2.69** |
|  | **Exponential** | 91 | 1 | - | 0.03 | 832.12 | **0.00** | **0.73** | **1.00** |
| 41 days | Normal | 46 | 2 | 41.32 | 38.24 | 470.05 | 33.60 | 0.00 | >1000 |
|  | Lognormal | 46 | 2 | 3.1 | 1.31 | 444.88 | 8.43 | 0.01 | 67.63 |
|  | **Gamma** | 46 | 2 | 0.94 | 0.02 | 438.51 | **2.06** | **0.26** | **2.80** |
|  | **Exponential** | 46 | 1 | - | 0.02 | 436.45 | **0.00** | **0.73** | **1.00** |
| 60 days | Normal | 46 | 2 | 55.7 | 56.49 | 505.96 | 42.04 | 0.00 | >1000 |
|  | Lognormal | 46 | 2 | 3.25 | 1.56 | 474.74 | 10.82 | 0.00 | >100 |
|  | **Gamma** | 46 | 2 | 0.78 | 0.01 | 463.95 | **0.03** | **0.50** | **1.01** |
|  | **Exponential** | 46 | 1 | - | 0.02 | 463.92 | **0.00** | **0.50** | **1.00** |
| 158 days | Normal | 32 | 2 | 81.57 | 79.27 | 375.08 | 27.46 | 0.00 | >1000 |
|  | Lognormal | 32 | 2 | 3.57 | 1.87 | 363.64 | 16.02 | 0.00 | >1000 |
|  | **Gamma** | 32 | 2 | 0.72 | 0.009 | 347.62 | **0.00** | **0.52** | **1.00** |
|  | **Exponential** | 32 | 1 | - | 0.012 | 347.82 | **0.20** | **0.47** | **1.10** |

**Figure S1.** Regressions of centroid size on fork length (upper panel; *R*^2^ = 0.99, *F*_1,210_ = 17726.54, *P* < 0.000001) and body mass (lower panel; *R*^2^ = 0.97, *F*_1,210_ = 7940.75, *P* < 0.000001) at the beginning of the experiment. All variables are log_10_-transformed.

**Figure S2.** Regressions of centroid size on fork length (upper panel; *R*^2^ = 0.99, *F*_1,30_ = 4716.24, *P* < 0.000001) and body mass (lower panel; *R*^2^ = 0.95, *F*_1,30_ = 601.57; *P* < 0.000001) at the end of the experiment. All variables are log_10_-transformed.

**Figure S3.** Linear relationships between spatial positions by days 41 and 60 (upper panel: *R*^2^= 0.97; *F*_1,21_= 615.45, *P*< 0.00001), 41 and 158 (middle panel: *R*^2^= 0.94; *F*_1,15_= 217.20, *P*< 0.00001), and 60 and 158 (lower panel: *R*^2^= 0.99; *F*_1,15_= 1199.13, *P*< 0.00001).
